# A *KCNQ4* c.546C>G Genetic Variant Associated with Late Onset Non-Syndromic Hearing Loss in a Taiwanese Population

**DOI:** 10.3390/genes12111711

**Published:** 2021-10-27

**Authors:** Ting-Ting Yen, I-Chieh Chen, Men-Wei Hua, Chia-Yi Wei, Kai-Hsiang Shih, Jui-Lin Li, Ching-Heng Lin, Tzu-Hung Hsiao, Yi-Ming Chen, Rong-San Jiang

**Affiliations:** 1 Department of Otolaryngology, Taichung Veterans General Hospital, Taichung 40705, Taiwan; tingting@vghtc.gov.tw (T.-T.Y.); ppmabcde@hotmail.com (M.-W.H.); dddd900901@hotmail.com (K.-H.S.); rayray0415@gmail.com (J.-L.L.); 2 School of Medicine, National Yang Ming Chiao Tung University, Taipei 11221, Taiwan; 3 Department of Medical Research, Taichung Veterans General Hospital, Taichung 40705, Taiwan; icchen@vghtc.gov.tw (I.-C.C.), feedfly210@hotmail.com (C.-Y.W.), epid@vghtc.gov.tw (C.-H.L.), thsiao@vghtc.gov.tw (T.-H.H.); 4 Department of Health Care Management, National Taipei University of Nursing and Health Sciences, Taipei 108306, Taiwan; 5 Department of Industrial Engineering and Enterprise Information, Tunghai University, Taichung 40704, Taiwan; 6 Department of Public Health, College of Medicine, Fu Jen Catholic University, New Taipei City 242062, Taiwan; 7 Institute of Public Health and Community Medicine Research Center, National Yang Ming Chiao Tung University, Taipei 11221, Taiwan; 8 Institute of Genomics and Bioinformatics, National Chung Hsing University, Taichung 402202, Taiwan; 9 Division of Allergy, Immunology and Rheumatology, Taichung Veterans General Hospital, Taichung 40705, Taiwan; 10 Rong Hsing Research Center for Translational Medicine & Ph.D. Program in Translational Medicine, Taichung 402202, Taiwan; 11 College of Medicine, National Chung Hsing University, Taichung 402202, Taiwan; 12 School of Medicine, Chung Shan Medical University, Taichung 40201, Taiwan

**Keywords:** autosomal dominant nonsyndromic hearing loss, *KCNQ4* c.546C>G, audiograms, pure tone audiometry

## Abstract

Clinical presentation is heterogeneous for autosomal dominant nonsyndromic hearing loss (ADNSHL). Variants of *KCNQ4* gene is a common genetic factor of ADNSHL. Few studies have investigated the association between hearing impairment and the variant c.546C>G of *KCNQ4*. Here, we investigated the phenotype and clinical manifestations of the *KCNQ4* variant. Study subjects were selected from the participants of the Taiwan Precision Medicine Initiative. In total, we enrolled 12 individuals with *KCNQ4* c.546C>G carriers and 107 non-carriers, and performed pure tone audiometry (PTA) test and phenome-wide association (PheWAS) analysis for the patients. We found that c.546C>G variant was related to an increased risk of hearing loss. All patients with c.546C>G variant were aged >65 years and had sensorineural and high frequency hearing loss. Of these patients, a third (66.7%) showed moderate and progressive hearing loss, 41.7% complained of tinnitus and 16.7% complained of vertigo. Additionally, we found a significant association between *KCNQ4* c.546C>G variant, aortic aneurysm, fracture of lower limb and polyneuropathy in diabetes. *KCNQ4* c.546C>G is likely a potentially pathogenic variant of ADNSHL in the elderly population. Genetic counseling, annual audiogram and early assistive listening device intervention are highly recommended to prevent profound hearing impairment in this patient group.

## 1. Introduction

Hearing loss is a common disability worldwide, involving inherited sensory defects. The global prevalence of hearing impairment in 2008 was 1.4% for children aged 5–14 years, 12.2% for males >15 years of age, and 9.8% for females >15 years of age [1]. Hearing loss is classified into three types: sensorineural, mixed, and conductive. Sensorineural hearing impairment (SNHL) involves pathological conditions of the inner ear. It occurs in about 1.9 out of 1000 live births and in 2% of school-age children. More than half of childhood SNHL cases likely have genetic etiology [2,3,4], and these are categorized as hereditary hearing impairments (HHIs).

Current reports show that 123 genes are pathogenically related to non-syndromic HHI [5]. HHI is classified based on onset time, prelingual or postlingual. A majority (80%) of prelingual and non-syndromic HHIs obey the autosomal recessive trait, 20% the autosomal dominant trait, and 1–1.5% X-linked, mitochondrial, or other inheritance patterns [6]. Post-lingual non-syndromic HHI commonly follows an autosomal dominant trait [7]. To date, 51 genes have been identified in the pathogenesis of autosomal dominant nonsyndromic hearing loss (ADNSHL) [5]. Current data suggest that mutations in *WFS1, KCNQ4, COCH,* and *GJB2* are more common as causes of ADNSHL [8]. Among them, *KCNQ4* accounts for 9% of ADNSHL cases [9]. The onset of hearing loss from the *KCNQ4* gene starts in the second decade and its audio profile presents high-frequency and progressive hearing loss [10].

*KCNQ4* is a voltage-activated potassium channel. Potassium recycling in the inner ear is an important process for maintaining auditory sensitivity. In the labyrinth, this process modulates how hair cells, such as the vestibular type I cell and cochlear outer hair cell, handle their responses to sensory stimulation, by exuding potassium back to the endolymph [11,12]. Spiral ganglion neuron death and inner hair cell death result in severe hearing loss, as shown in the animal study [13].

Currently, of the 38 identified variants of *KCNQ4*, 30 of them are inherited via missense mutation [14]. There are 17 known pathogenic variants from the ClinVar database [15]. The main pathologic variants are missense mutations, with symptom onset invariably during childhood. Variant c.827G>C is the most common and was first reported by Dutch and Japanese scientists. For another two deletion mutations (c.211_223del13 and c.211delC), symptoms commence during adolescence [16,17]. In addition, in Taiwan, two silent mutations of uncertain clinical significance have been reported. Variants such as c.648C>T and c.1503C>A also have an onset of symptoms during childhood [18]. Another study from a public database in Korea revealed 17 rare loss-of-function and six missense variants of *KCNQ4*; these variants are likely related to late-onset hearing loss [19]. One study in Taiwan on nonsyndromic deafness reported three variants of *KCNQ4* (c.546C>G, c.648C>G, and c.1503C>T) from three carriers. The carrier for c.546C>G exhibits prelingual and profound deafness [18,20]. However, no large-scale study has been conducted regarding the role of c.546C>G in non-syndromic deafness.

Here, we aimed to explore the possible contribution of the c.546C>G variant of the *KCNQ4* gene to post-lingual non-syndromic HHIs in a hospital-based population in Taiwan.

## 2. Materials and Methods

### 2.1. Data Source

This case-control study was conducted using data from the Taiwan Precision Medicine Initiative (TPMI), which gathered information and specimens from a convenience sample of Taiwanese volunteer participants in Taichung Veterans General Hospital (TCVGH) during the period from June 2019 to June 2020. Between June 2019 to June 2020, patients aged >18 years who visited 28 medical and surgical outpatient clinics in Taichung Veterans General Hospital (TCVGH) were invited to participate in the study. Our study cohort consisted of 32,728 patients (mean age 57.3 ± 15.1 years, 15,249 male, 17,479 female) with genotyping information, demographics, and medical history, as well as biochemical reports, and all of the participants provided informed consent. The study was conducted in accordance with the Declaration of Helsinki, and the study protocol was approved by the Institutional Review Board of TCVGH (SF19153A). This case-control study followed the Strengthening the Reporting of Observational Studies in Epidemiology (STROBE) reporting guidelines.

### 2.2. Participants

As illustrated in Supplementary Figure S1, the sample base included participants who had audiograms recorded in the Department of Otorhinolaryngology in TCVGH between June 2019 and June 2020. We extracted participants aged >18 years who had received pure tone audiometry (PTA). The data source contained a total of 527 participants who received PTA, 179 of whom had no available audiogram records and were thus excluded. The final study population included 348 patients with available audiograms, of which, 236 had sensorineural/mixed hearing loss (MHL) and were designated as the case group. Among the 112 participants who did not have SNHL/MHL, 5 of them were excluded for mild conductive hearing loss (CHL). Accordingly, the remaining 107 participants without the *KCNQ4* c.546C>G variant and with audiograms were assigned to the control group. We further analyzed the association between the *KCNQ4* c.546C>G variant and non-syndromic hearing loss. In the SNHL/MHL group, 12 patients had the *KCNQ4* c.546C>G variant and were defined as *KCNQ4* c.546C>G variant carriers, and the remaining 224 patients who did not have *KCNQ4* c.546C>G variant were defined as *KCNQ4* c.546C>G variant non-carriers.

### 2.3. Pure Tone Audiometry (PTA)

Audiometric tests were conducted with a pure tone audiometer (Grason Stadler, Otometrics, USA). Pure tone bone and air conduction hearing thresholds were assessed according to standard procedures at frequencies of 250, 500, 1000, 2000, 4000, and 8000 Hz. Pure tone averages (PTA) were ciphered from frequencies of 500, 1000, 2000, and 4000 Hz. The level of the individual subject’s hearing loss was graded based on disparity levels against PTA, as normal hearing (<20 dB), mild hearing loss (21–40 dB), moderate hearing loss (41–70 dB), severe hearing loss (71–95 dB), or profound hearing loss (>95 dB). Hearing loss was typed as SNHL, mixed hearing loss, or conductive type hearing loss according to the patterns in pure-tone air- and bone-conduction thresholds. Sensorineural and mixed types of hearing loss were defined as hearing loss in this study. High-frequency hearing loss was defined as hearing loss in the range between 2000 and 8000 Hz.

### 2.4. Phenome-Wide Association Studies (PheWAS)

Clinical diagnoses of c.546C>G carriers and non-carriers were extracted from TCVGH electronic health records according to the diagnostic codes of the International Classification of Diseases, Ninth Revision (ICD-9). Phenotypes were detected by guiding ICD-9 codes and separated disease entities. Patients were defined to have a certain phenotype if they had the matching ICD diagnosis on one or more dates, while phenotypic controls consisted of those participants who had never matched the ICD code. The PheWAS Manhattan plot was used to correlate phenotype with genotype. Such plots revealed any significant association between different phenotypes of the variant c.546C>G. On the x-axis, we display different disease groups in different colors. The y-axis shows the *p* values of individual phenotypes. Blue and red horizontal lines mark *p* values at 0.05 and 0.001. A Quantile–Quantile (Q–Q) plot was used to evaluate whether the observed distribution was different from the expected distribution under the null hypothesis (Figure 2).

### 2.5. Genetic Analysis 

Taiwan Biobank version 2 (TWBv2) (Affymetrix, Santa Clara, CA, USA) array adopted the whole-genome sequence (WGS) data from Taiwan Biobank (TWB) participants to select single nucleotide polymorphisms (SNPs) best for imputation in Han Chinese samples in Taiwan, enclosed 114,000 risk variants in 2831 unusual disease genes chosen from the ClinVar, ACMG, HGMD, GWAS Catalog, locus-specific databases and published literature [21]. We genotyped 32,728 TPMI participants using the TWBv2 array, and then normalized the TWB reference panel to calculate minor allele frequency (MAF) of all hearing loss related SNPs.

### 2.6. Statistical Analysis

The basic characteristics of hearing loss-associated variants in the TPMI were described in number and minor allele frequencies. Fisher’s exact and chi-square tests were applied to compare categorical variables between case and control groups. Univariable logistic regression was used to evaluate the odds ratio (OR) of the *KCNQ4* c.546C>G variant for hearing loss. Statistical analyses were evaluated with Windows SPSS Statistics (Armonk, NY, USA: IBM Corp.), version 22. *p* values < 0.05 were considered statistically significant.

## 3. Results

### 3.1. Genetic Variants Associated with Hearing Loss 

Between June 2019 and June 2020, the TPMI identified 32,728 patients (15,249 men and 17,479 women, with a mean age of 57.31 ± 15.05, who were recruited from 28 medical and surgical outpatient clinics) from TCVGH with genotyping microarrays. They all had 11 identified risk variants in seven rare diseases related to hearing loss based on the TWBv2 array (Table 1). The minor allele frequency (MAF) of *KCNQ4* c.546C>G was 0.61% at TCVGH, which was higher than that of the East Asian population in the GnomAD database. The MAFs of the *SLC26A4* c.919-2A>G, *GJB2* c.299_300del, and MT-RNR1 rs28358569 were 0.78%, 0.1%, and 4.49%, respectively, and were similarly higher than that of the East Asian population. On the other hand, the MAFs of *SLC26A4* c.2168A>G and *GJB2* c.235del were 0.09% and 0.59%, respectively, and were low compared with those of the East Asian population. No compound heterozygotes were observed in our cohort. These results suggested that *KCNQ4* c.546C>G was the most common variant among those affected by ADNSHL in the Taiwanese population.

### 3.2. Patient Characteristics and Audiograms 

In this study, we identified 348 patients with available audiograms, 236 of whom had SNHL/MHLand were designated as thecase group. The 107 participants without SNHL/MHL wereassigned to thecontrol group (Table 2). The demographic characteristics are reported by categories of age, gender, hearing status, SNPs and comorbidity. The prevalence of hearing loss, *KCNQ4* c.546C>G variant, and hypertension were all significantly higher in the case group.

In the case group, a total of 12 patients with the *KCNQ4* c.546C>G variant (Supplementary Figure S1) were classified as carriers; the other 224 patients were defined as non-carriers. The carrier group included six men and six women with a mean age of 72.4 years (range: 66 to 78 years), as shown in Table 3. They were elderly patients (≥65 years old) when receiving their first hearing tests at TCVGH. All patients had SNHL with high-frequency hearing loss. Hearing loss was bilateral in 10 patients (83.3%). Progressive hearing loss was found in eight patients (66.7%), tinnitus in five patients (41.7%), and vertigo in two patients (16.7%). The severity of hearing loss ranged from mild to profound (with 16.7% mild, 66.7% moderate, and 8.3% severe and profound) (Table 3). These results indicated that old age and moderate hearing loss were significantly different between groups. 

**Figure 1:**
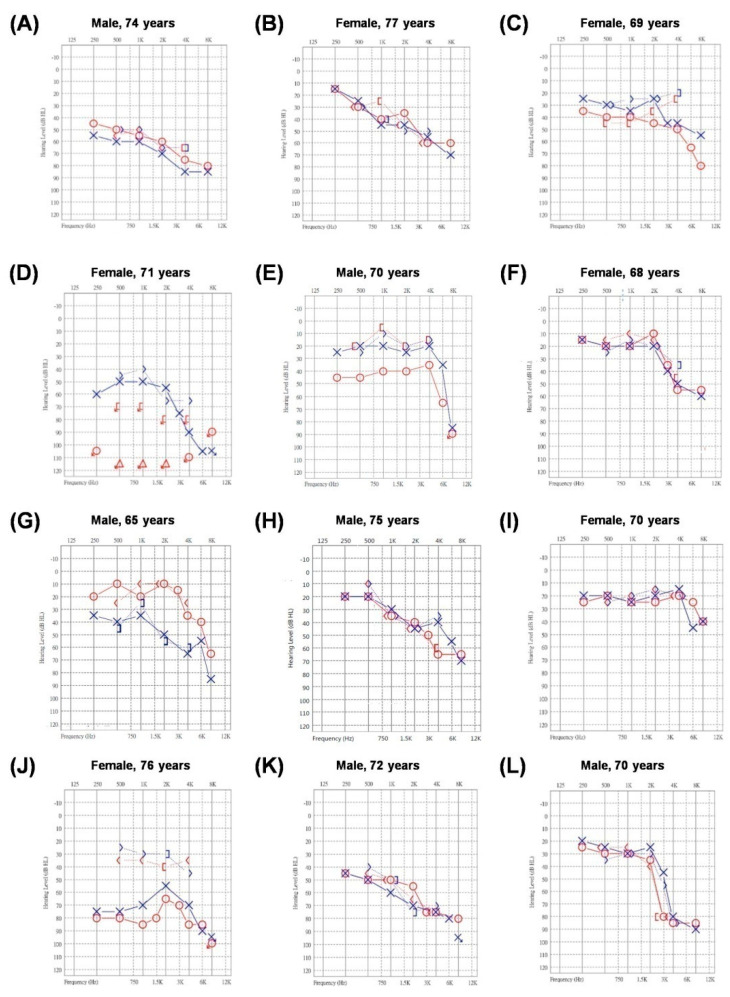
(**A**–**L**) Age, sex, and audiograms of twelve patients with the *KCNQ4* c.546C>G variant.

Figure 1 shows the pure tone audiograms of the 12 patients who had SNHL/MHL with high frequency hearing loss. 11 patients had SNHL and one patient had MHL (Figure 1J). Most of them exhibited similar features of hearing impairment that was bilaterally symmetric, moderate to profound with flat configurations. Only one patient had the co-existing *GJB2* (c.235del) heterozygous variant (Figure 1A).

Right ear air conductions displayed as circles connected by lines, and left ear air conductions are represented denoted by ×’s connected by lines.

The *KCNQ4* c.546C>G variant group consisted of elderly patients with a mean age of 72.4 years. They were compared with patients aged ≥ 70 years with SNHL/MHL to evaluate the association of the *KCNQ4* c.546C>G variant and age with non-syndromic hearing loss. There were 10 carriers and 78 non-carriers who were 70 years or older when the data was recorded from the otorhinolaryngological tests. In Table 4, univariable logistic regression analysis reveals that the *KCNQ4* c.546C>G variant was associated with a numerically higher risk of moderate hearing loss (OR, 4.20; 95% CI, 0.49-36.26; *p* = 0.43) in patients aged over 70.

### 3.3. PheWAS of KCNQ4 c.546C>G Variant and Clinical Diagnosis

**Figure 2:**
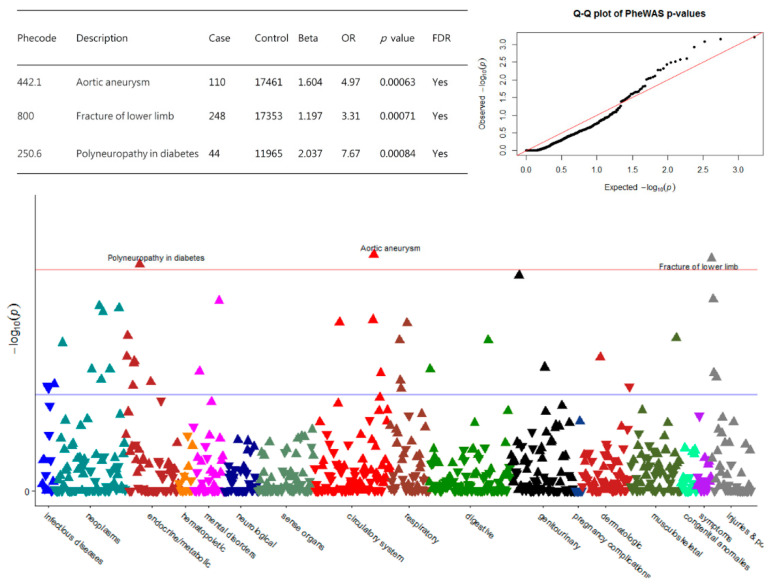
The Manhattan and Q-Q plots of association between *KCNQ4* c.546C>G and disease outcomes among 32,728 participants from the study cohort.

Figure 2 shows the PheWAS Manhattan and Q-Q plots for *KCNQ4* c.546C>G and clinical diagnosis identified in the electronic health records. We found an association between *KCNQ4* and aortic aneurysm (OR: 4.97, *p* = 6.3 × 10^−4^), fracture of lower limb (OR: 3.31, *p* = 7.1 × 10^−4^) and polyneuropathy in diabetes (OR: 7.67, *p* = 8.4 × 10^−4^). The Q-Q plot of the observed versus expected p-values that showed clear enrichment of p-values in Figure 2. Thus, we could expect the TPMI data to be suitable for the PheWAS of the *KCNQ4* c.546C>G, and we had sufficient power to detect modest association signals for diseases.

Refer to table for number of cases and controls per genotype, OR (95% CI), and *p* value (logistic regression) for each PheWAS signal. False discovery rate (FDR) corrected significant threshold (*p* < 0.001) is represented by a red line. The blue horizontal line is displayed as the significance level of *p* = 0.05.

## 4. Discussion

In this hospital-based real-world study of an elderly Taiwanese population, we found that the *KCNQ4* c.546C>G variant was associated with a four-fold increased risk for SNHL and high-frequency hearing loss. We showed that genetic factors likely contributed to age-related hearing loss. We also explored the associations between a hearing loss-related genetic variant and clinical phenotypes of different disease entities. Our results suggested that annual hearing check-ups could be beneficial for *KCNQ4* c.546C>G carriers. Genetic evaluations might provide additional information for patients with age-related hearing loss to guide early intervention with assistive listening devices.

The *KCNQ4* c.546C>G variant is a missense mutation (F182L), which is located in the S3 domain of *KCNQ4* and is highly conserved in *KCNQ4* among various species. *KCNQ4* encodes a protein that constitutes a potassium channel. The potassium channel has a P-loop region forming the channel pore and six transmembrane domains. The glycine–tyrosine–glycine sequence within the P-loop encompasses the selectivity filter distinguishing potassium ions for transport needs [22]. The animal study performed by Su et al. [18,20] investigated *KCNQ4* channels in *Xenopus* oocytes, and measurements were performed using a two-electrode voltage clamp. The *KCNQ4* outward current induced by the calcium salt, ionomycin, was significantly increased (about 1.7-fold) from the control current amplitude of +60 mV. Additionally, mutation F182L of the *KCNQ4* gene was investigated in the subcellular localization of HeLa cells, which resulted in proteins with impaired trafficking, localized in perinuclear vesicles, but not localized to the membrane [18,20]. 

In Taiwan, two silent mutations of uncertain clinical significance were reported. Variants such as *KCNQ4* c.648C>T and *KCNQ4* c.1503C>A both cause protein changes (R216R and T501T, respectively) and the onset of symptoms during childhood. Su et al. [18] discovered that the silent mutation, R216R, does not alter the content of amino acid residue. However, the neural network prediction system revealed that it could potentially create a novel splice donor site during transcription, and lead to a loss of 66 nucleotides, which might affect the protein structure of *KCNQ4* and the function of the K+ channel. On the contrary, the T501T mutant results in a protein that is localized to the membrane, similar to a normal *KCNQ4* protein. Therefore, the mutation T501T of the *KCNQ4* gene did not affect the synthesis of *KCNQ4* proteins, nor would their transportation to the cell membrane to form functional potassium channels. The role of the T501T mutant in hereditary hearing loss requires further investigation to evaluate its function in the *DFNA2/KCNQ4* gene.

To judge the pathogenicity of the *KCNQ4* c.546C>G variant, CADD scores were taken from the “CADD” project website (version v1.5) [23]. The value of the CADD Raw score is 2.205. The positive value indicates that the *KCNQ4* c.546C>G variant is more likely to be stimulated and have deleterious effects based on different genomic features. A CADD Phred-scaled score of 20.9 means that the variant is potentially pathogenic, with a cutoff value of 15. According to the ACMG guidelines and the Annovar database, the *KCNQ4* c.546C>G variant is classified as 2PM (pathogenic moderate) and 1PP (pathogenic supporting) evidence [24]. Meanwhile, our report demonstrated an increased risk in patients with the *KCNQ4* c.546C>G variant. Although the risk did not reach statistical significance, we did observe SNHL in multiple unrelated patients with the c.546C>G variant. Therefore, our study provided further evidence of moderate pathogenicity. Taken together, we judge the *KCNQ4* c.546C>G variant as 3PM and 1PP, i.e., likely pathogenic, by ACMG guidelines.

Most *KCNQ4* pathogenic variants are missense alterations, and the phenotype reflects the effect of faulty proteins in the inner ear [25]. Some pathogenic variants directly involve the selectivity of the filter, therefore causing a progressive loss in potassium recycling within the inner ear, and compromising auditory function over time [22]. Currently, all the main pathologic variants of *KCNQ4* are missense variants, affecting the P-loop protein domain, and the onset of symptoms appears during childhood [17]. However, a prior study demonstrated that the c.546C>G variant yielded similar cell surface expression and functional features to a wild-type channel current [17]. We are the first to report on c.546C>G variant carriers who have presented with postlingual and moderate hearing loss. All participating c.546C>G carriers were >65 years of age and had no complaints of hearing impairment during childhood. Further prospective studies are needed to elucidate the underlying pathogenic mechanisms and clinical implications.

In the elderly, age-related hearing loss is the most common sensory loss. Noise exposure, metabolic stress, and genetic propensities are causes of age-related hearing loss [26]. Both hereditary and noise-induced hearing loss are associated with declined cell surface expression and damaged potassium channels in outer hair cells [26]. One genetic variant, c.777T>C in *KCNQ4*, results in a higher risk of noise-induced hearing loss for noise-exposed Chinese workers [27]. Our findings were consistent with a previous study which showed that the *KCNQ4* gene is likely related to the complex interactions between age, environmental noise, and hearing loss. Collectively, our findings suggest that carriers of all variants of the *KCNQ4* gene should avoid noise exposure to prevent further damage to the inner ear.

In the present study, the PheWAS data implied an association of the *KCNQ4* variant with aortic aneurysm and diabetic polyneuropathy. An animal study in rats reported that the *KCNQ4*-encoded voltage-dependent potassium (Kv7.4) channel contributes to the β-adrenoceptor-mediated vasodilatation of smooth muscles in renal arteries [28]. Down-regulation of the Kv7.4 channel may be critical in the development of hypertension [28]. Since common risk factors of aortic aneurysm and diabetic polyneuropathy include old age, hypertension, hyperlipidemia, and smoking [29,30,31,32], we speculate that an alternative Kv7.4 channel likely contributes to hypertension and poor diabetes control, finally leading to aortic aneurysm and polyneuropathy. Further studies are required to clarify the vessel-specific effects of hypertension on the Kv7.4 channel.

In addition, this study demonstrated the correlation between the *KCNQ4* variant and the fracture of lower limbs. In a prior family study, no vestibular problem was observed in families with pathogenic variants of *KCNQ4* [33]. Our results indicated that 16.7% of the c.546C>G variant carriers had previously sought medical treatment due to vertigo. In a previous Dutch study, a hyperactive vestibular-ocular reflex was found in 30% of individuals with pathogenic variants of *KCNQ4* [34]. Hearing loss was also reported to be an independent factor for self-reported falls in the elderly population [35]. With a hearing loss of 10 dB, the risk of falls increases by 40% [35]. We hypothesize that the co-existing vestibular dysfunction due to the c.546C>G variant contributes to falls, and subsequently to lower limb fractures. Future prospective studies on the vestibular functions of *KCNQ4* c.546C>G carriers are needed to confirm our hypothesis.

Our study has several limitations, despite being based on real-world data from patients with hearing loss. Firstly, we used the SNP array for genetic profiling. Not every variant of hearing loss was included in the probe design [21]. Therefore, we cannot exclude the potential effects of co-existing hearing loss variants in the study group. Sequencing data is needed to unravel genetic contributions to hearing loss [36]. Secondly, the study design is retrospective and the number of participants with audiograms was limited. The onset of hearing loss, the severity of hearing loss, family pedigrees, or noise exposure were not prospectively collected. Nevertheless, this study involves the largest patient group with *KCNQ4* c.546C>G carriers in a hospital-based population. An association of *KCNQ4* c.546C>G with hearing loss in an elderly population was established. Lastly, only patients with blood tests participated in the TPMI study. The presence of selection bias is likely, underestimating minor allele frequencies.

## 5. Conclusions

In summary, this hospital-based study demonstrated that the c.546C>G variant of the *KCNQ4* gene is associated with postlingual hearing loss in an elderly population in Taiwan. Genetic factors might be involved in age-related hearing loss. Our data suggested that genetic tests for the *KCNQ4* c.546C>G variant are necessary for patients with age-related hearing loss or indecipherable postlingual hearing loss. Early family genetic counseling, annual audiograms, and early assistive listening device interventions are crucial to prevent profound hearing impairments in this group of patients.

## Figures and Tables

**Table 1:** Genetic variants associated with hearing loss among the 32,728 participants from the TPMI.

CHR	SNP	Gene	Locus	Homozygote Genotype/Homoplasmic mtDNA Mutation	Heterozygote Genotype/Heteroplasmic mtDNA Mutation	MAF in TCVGH	MAF in GnomAD/HGDP-CEPH-db Supplement 1 (Population: East Asian)	Phenotype & Inheritance Mode
1	rs80358273	*KCNQ4*	DFNA2A	1	398	0.61%	0.00%	Nonsyndromic hearing loss, AD
	c.546C>G							
4	rs387906930	*WFS1*	DFNA6	0	79	0.12%	0.00%	Wolfram-like syndrome, AD
	c.2051C>T							
7	rs111033313	*SLC26A4*	DFNB4	0	516	0.78%	0.33%	Pendred syndrome, AR
	c.919-2A>G		Pendred					
7	rs121908362	*SLC26A4*	DFNB4	0	59	0.09%	0.22%	Pendred syndrome, AR
	c.2168A>G		Pendred					
9	rs745750156	*CEP78*		0	59	0.09%	0.00%	Cone-rod dystrophy and hearing loss 1, AR
	c.1251+5G>A							
13	rs111033204	*GJB2*	DFNB1A	0	67	0.10%	0.00%	Nonsyndromic hearing loss, AR
	c.299_300del							
13	rs80338943	*GJB2*	DFNB1A	2	388	0.59%	2.70%	Nonsyndromic hearing loss, AR
	c.235del							
MT	rs121434453	*MT-TE*		37	0	0.12%	None	Diabetes-deafness syndrome
	m.14709T>C							
MT	rs267606617	*MT-RNR1*	12rRNA	44	0	0.13%	None	Aminoglycoside-induced deafness
	m.1555A>G							
MT	rs267606618	*MT-RNR1*	12rRNA	72	0	0.22%	None	Aminoglycoside-induced deafness
	m.1095T>C							
MT	rs28358569	*MT-RNR1*	12rRNA	1476	0	4.49%	0.40%	Aminoglycoside-induced deafness
	m.827A>G							

Abbreviation: CHR, chromosome; mtDNA, mitochondrial DNA; MAF, minor allele frequency; TPMI, Taiwan Precision Medicine Initiative; TCVGH, Taichung Veterans General Hospital; AD, autosomal dominant; AR, autosomal recessive.

**Table 2:** Basic characteristics of study populations.

Variables ^a^	Case Group		Control Group		*p* Value ^b^
	(SNHL/MHL *n* = 236)		(non SNHL/MHL *n* = 107)		
	*n*	%	*n*	%	
Age (years)					<0.0001
18–64	41	17.37	65	60.75	
≥65	195	82.63	42	39.25	
Gender					0.003
Male	140	59.3	45	42.06	
Female	96	40.7	62	57.9	
Hearing status					<0.0001
Normal <20 dB	0	0.0	59	55.1	
Mild 20–40 dB	70	29.7	48	44.9	
Moderate 40–70 dB	119	50.4	0	0.0	
Severe 70–95 dB	30	12.7	0	0.0	
Profound >95 dB	17	7.2	0	0.0	
SNPs					
*KCNQ4* c.546C>G	12	5.1	0	0.0	0.02
*WFS1* c.2051C>T	0	0.0	0	0.0	
*SLC26A4* c.919-2A>G	3	1.3	3	2.8	0.38
*SLC26A4* c.2168A>G	0	0.0	0	0.0	
*CEP78* c.1251+5G>A	1	0.4	1	0.9	0.53
*GJB2* c.299_300del	0	0.0	0	0.0	
*GJB2* c.235del	4	1.7	0	0.0	0.31
Comorbidities					
Hyperlipidemia	162	68.6	73	68.2	0.94
Hypertension	183	77.5	65	60.8	0.001
Diabetes mellitus	159	67.4	67	62.6	0.39
Coronary artery disease	93	39.4	36	33.6	0.31
Chronic Kidney Disease	143	60.6	48	44.9	0.007

^a^ Categorical variables are expressed as numbers (percent); ^b^ Comparison of categorical variables was analyzed using the Fisher’s exact and chi-square tests. Abbreviations: SNHL, sensorineural hearing loss; MHL, mixed hearing loss.

**Table 3:** Characteristics of *KCNQ4* c.546C>G carriers and non-carriers.

Variables ^a^	Carriers (*n* = 12)		Non-Carriers (*n* = 224)	
	*n*	%	*n*	%
Age (years)				
18–64	0	0	41	18.3
≥65	12	100	183	81.7
Gender				
Male	6	50.0	90	40.18
Female	6	50.0	134	59.8
Hearing status				
Normal <20 dB	0	0.0	0	0.0
Mild 20–40 dB	2	16.7	68	30.4
Moderate 40–70 dB	8	66.7	111	49.6
Severe 70–95 dB	1	8.3	29	13.0
Profound >95 dB	1	8.3	16	7.1
Comorbidities				
Hyperlipidemia	8	66.7	154	68.8
Hypertension	9	75.0	174	77.7
Diabetes mellitus	8	66.7	151	67.4
Coronary artery disease	5	41.7	88	39.3
Chronic Kidney Disease	10	83.3	133	59.4

^a^ Categorical variables are expressed as numbers (percent).

**Table 4:** Association of hearing loss and *KCNQ4* c.546C>G variant in patients aged over 70.

Variable	OR	95% CI		*p* Value ^a^
**Hearing status (ref = Mild 20–39 dB)**				
Moderate 40–69 dB	4.20	0.49	36.26	0.43
Severe 70–95 dB	2.67	0.15	47.30	0.99
Profound >95 dB	4.80	0.26	90.30	0.53

^a^ Univariable logistic regression analysis was used to compare variable between the carriers and non-carriers. Abbreviation: OR, odds ratio; 95% CI, 95% confidence interval.

## Data Availability

The clinical data presented in this study are available on request from the corresponding authors. The genetic data from the Taiwan Precision Medicine Initiative are not publicly available. The authors confirm that, for approved reasons, some access restrictions may apply to the data underlying the findings. The data used in this study cannot be made available in the manuscript, the supplemental files, or in a public repository due to the Personal Information Protection Act executed by Taiwan’s government, starting in 2012. Requests for data can be sent as a formal proposal to obtain approval from the ethics review committee of the appropriate governmental department in Taiwan.

## Supplementary Materials

The following are available online at https://www.mdpi.com/article/10.3390/genes12111711/s1, Figure S1: Flowchart illustrating the study design.
