# Supplementary figures and images for: A KCNQ4 c.546C>G Genetic Variant Associated with Late Onset Non-Syndromic Hearing Loss in a Taiwanese Population
Source: Genes (Basel). 2021 Oct 27;12(11):1711. doi: 10.3390/genes12111711 (PMC8618107; doi:10.3390/genes12111711)

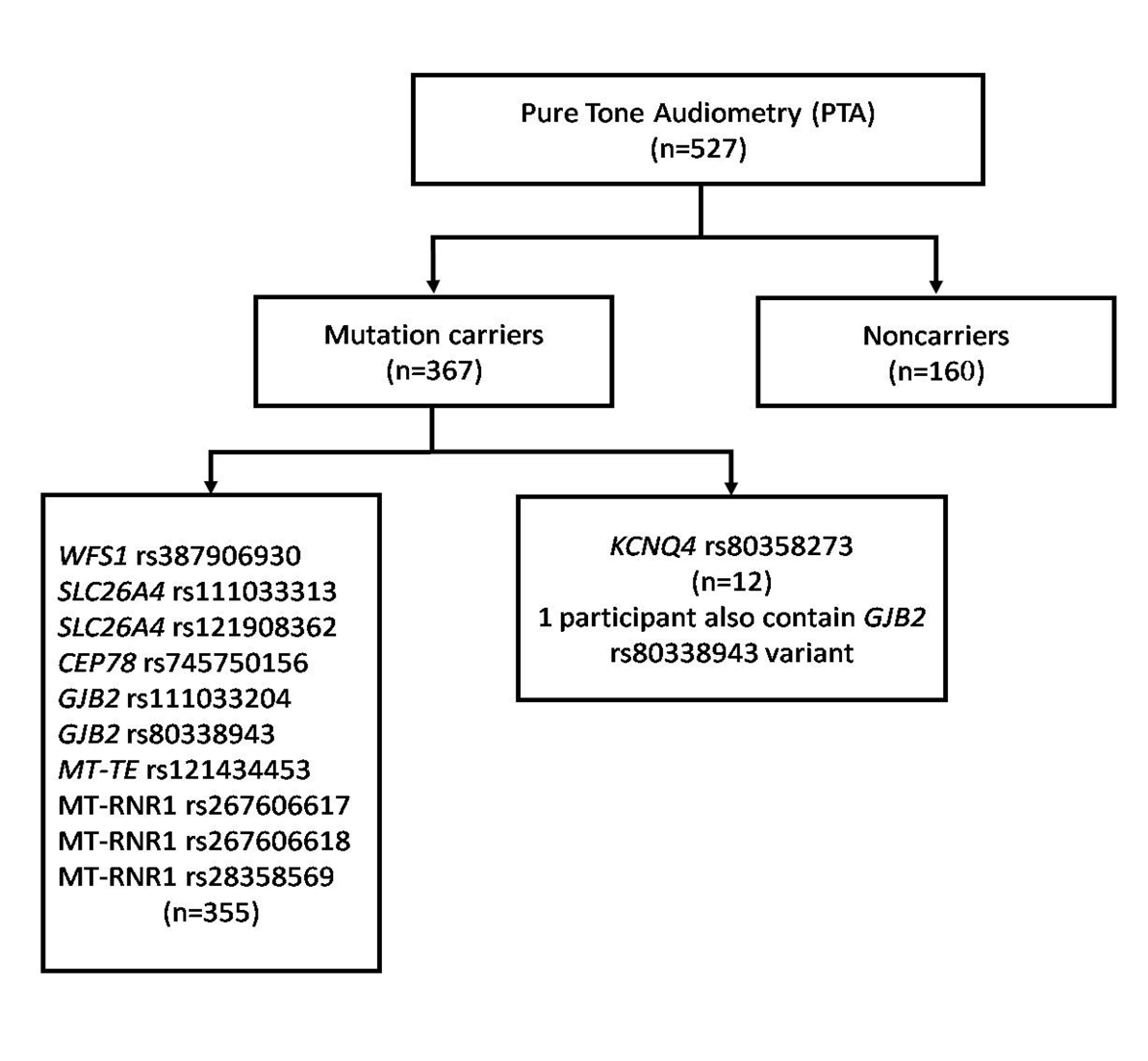

Supplement: Supplementary file 1 [file genes-12-01711-s001.zip › genes-1408258-supplementary.tif]
